# Immunomodulatory effects of novel nano micelle based curcumin in rheumatoid arthritis patients: A double blind randomized clinical trial

**DOI:** 10.1515/rir-2024-0031

**Published:** 2025-01-09

**Authors:** Faezeh Khamar, Mahdi Atabaki, Morteza Samadi, Marzieh Reisi, Mahnaz Sandoughi

**Affiliations:** Department of Immunology, Faculty of Medicine, Shahid Sadoughi University of Medical Sciences and Health Services, Yazd, Iran; Clinical Immunology Research Center, Zahedan University of Medical Sciences, Zahedan, Iran; Department of Immunology, School of Medicine, Isfahan University of Medical Sciences, Isfahan, Iran; Rheumatologis, Department of Internal Medicine, Ali Ebn Abitaleb Hospital, Zahedan University of Medical Sciences, Zahedan, Iran

**Keywords:** rheumatoid arthritis, curcumin, T helper

## Abstract

**Background and Objectives:**

Rheumatoid arthritis (RA) is a well-known systemic autoimmune inflammatory disease. This investigation aimed to assess the effects of Sina-curcumin, a novel nano micelle-based curcumin, on immune system responses of RA patients.

**Methods:**

This pilot study is a randomized double blinded, controlled trial. Patients who fulfilled the European League against Rheumatism-American College of Rheumatology (EULAR-ACR) criteria for RA were assigned to receive curcumin or placebo for 12 weeks. The outcomes of this study were comparison of changes in mean value of Disease Activity Score of 28 joints erythrocyte sedimentation rate (DAS28-ESR), erythrocyte sedimentation rate (ESR), C-reactive protein (CRP), frequency of T helper 1 and T helper 2 cells population.

**Results:**

From 150 RA patients who were assessed for eligibility, data from 30 patients (15 patients in each group) were analyzed. There was no significant difference between the two groups regarding age (*P* = 0.6441) and body mass index (BMI, *P* = 0.6016). Our measurement showed a statistically significant reduction in ESR (*P* < 0.0001), CRP (*P* < 0.0001) and a non-significant decrease in DAS28-ESR (*P* = 0.5125) in the curcumin group. Also, the Th1/Th2 ratio favorably decreased in the curcumin group. This finding was due to a significant increase in Th2 cells (*P* < 0.0001) and a nonsignificant decrease in Th1 cells (*P* = 0.1532).

**Conclusion:**

Our trial findings revealed the immunomodulatory effects of curcumin. It could be used and recommended as adjunctive treatment for RA patients.

## Introduction

Rheumatoid arthritis is a persistent, chronic autoimmune disorder that characterized by symmetrical small joint inflammation and varying degrees of damage to bones and cartilage leads to joint pain and disability.^[1]^ Rheumatoid arthritis (RA) affects nearly 1% of the population,^[2]^ and it is more common in women.^[3]^

Genetic elements, environmental factors, and immune responses are significantly involved in the development of RA.^[4]^ T cell activation triggers RA, and B cells amplify inflammation.^[5]^ There is imbalance between pro-inflammatory and anti-inflammatory cytokines, alongside the disruption of Th1/ Th2 and Th17/Treg cell equilibrium, further participates in the development of RA.^[4]^

There is currently no definite treatment for RA.^[6]^ Nonetheless, disease-modifying anti-rheumatic drugs (DMARDs), such as methotrexate have a significant effect in improving symptoms and preventing joints destruction.^[7]^ However, prolonged usage and limited target specificity of these drugs lead to adverse side effects, as well as imposing a substantial economic burden on those with RA.^[2,3,4,5]^ Given these challenges, there is an urgent need to find a safe and novel drug.

Curcumin, a phenolic compound^[7]^ derived from spice turmeric, boasts natural properties.^[8,9]^ Recent studies highlight curcumin’s anti-inflammatory, antioxidant, and immunoregulatory effects.^[5,10]^ Curcumin’s immunomodulatory effects arise from inhibiting pro-inflammatory Th17 cells, which shift the pro-inflammatory Th1 cells response to an anti-inflammatory Th2 cells response, enhance immunosuppressive Treg cells production, and suppress osteoclast activity.^[2,4,5]^ According to the United States Food and Drug Administration (FDA), the daily dosage of curcumin for maximal effectiveness is from 0 to 4 milligrams/kg.^[2]^ However, using curcumin as a therapeutic agent has limitations, including its low water solubility (hydrophobic nature), rapid metabolism, unstable chemical structure, poor intestinal absorption, and limited bioavailability.^[7,8,9]^ Fortunately, nano-formulated curcumin holds the potential to enhance solubility, stability, and absorption.

Curcumin has been administered at different doses in animal models and in human clinical trials which could be relieve pain and inflammation.^[11,12,13,14,15]^ However, there are few studies on the effectiveness of curcumin nanoformulation technology for treatment of RA patients.^[16,17]^ To the best of our knowledge, our present study is the first clinical trial designed to assess a nano formulated form of curcumin administration on immune cells in RA patients. The primary objective of our study involves evaluating the impact of Sina-curcumin, a novel curcumin-based nano micelle, on T-cells function and disease activity in RA patients.

## Materials and Methods

### Study Design and Participant Selection

**Figure 1:**
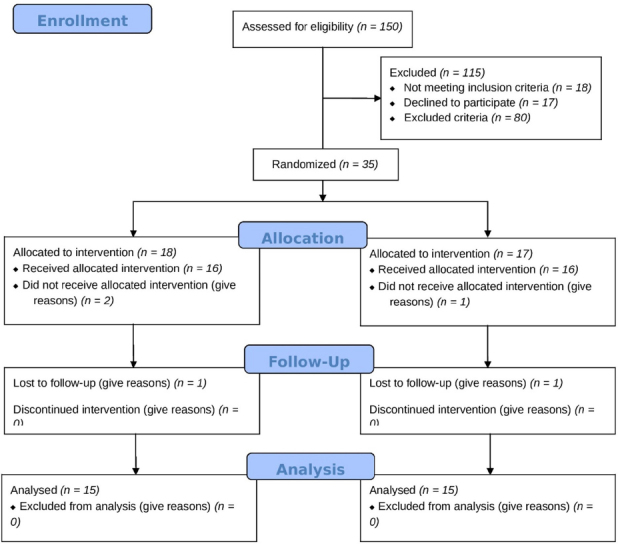
CONSORT flow diagram.

Participants were selected based on the European League Against Rheumatism-American College of Rheumatology (EULAR-ACR) criteria for RA. The study was conducted in a rheumatology clinic in Ali Ebn Abitaleb Hospital in Zahedan, the capital of Sistan and Baluchestan Province in Iran. The duration of the study was from August 2022 to April 2023. Inclusion criteria were women RA patients with active disease defined as a Disease Activity Score of 28 joints (DAS28) score ≥ 2.6- < 5 and the age between 18–65 years old. Exclusions criteria were other autoimmune and chronic disease, pregnancy, lactation, psychiatric disorders, history of allergy and useage of more than 7.5 mg prednisolon daily and aspirin (ASA). The study adhered to the guidelines outlined in the Consolidated Standards of Reporting Trials (CONSORT) checklist, that depicted in Figure 1.

### Ethical Considerations

The study received ethical approval from the Ethical Committee of the Faculty of Medicine at Shahid Sadoughi University of Medical Sciences (Yazd, Iran), and the approval code was IR.SSU.MEDICINE.REC.1400.402. Our trial was also registered with the Iranian Registry of Clinical Trials (IRCT) under the registration number IRCT20 220 306 054 203N1. All participants provided informed consent after being fully informed.

### Patient Randomization

Patients were assigned to two groups using a permutation block stratified randomization method. The categorization process was initiated based on age brackets (18 to 40 and 41 to 65 years), and DAS28 score ranges (2.7 to 3.9 and 4.0 to 5.0) to achieve alignment.

### Allocation

The curcumin capsules (marketed under the trade name Sina Curcumin) and placebo capsuls (lactose) produced by Exir Nano Sina company in Tehran, Iran (IRC: 1228225765), were administered at a daily dosage of 40 mg. Identical placebo and curcumin capsuls prepared to blinding the study.

### Administration

Assessment of disease activity, liver and renal function test were done at baseline and 12 weeks after intervention by a rheumatologist who was blind to the allocations. Moreover, the percentage of Th1 and Th2 cells obtained by flow cytometry was performed by a researcher who was blind. Drug and placebo were administrated by a reasercher who do not involve in assessment of study outcoms. Patients were monitored by telephone contact at 4th and 8th weeks to ensure compliance. Adverse events were noted during the study. If there was significant adverse reaction, the patient was excluded from the study. All participants received their routine treatment beside the intervention or placebo.

### Disease Activity, C-reactive protein (CRP) and erythrocyte sedimentation rate (ESR) Measurement

DAS28-ESR, a validated composite disease activity score, which includs ESR, patient assessed global health score (scale of 0 to 100), swollen and tender joint counts (both 0–28) is used for assessment of disease activity. Higher score is representative of a worse situation.

Additionally, CRP levels were quantified using the nephelometry technique with a MININEPH™ kit from Binding Site (UK). The ESR was determined using the Westergren method.

### Blood Collection Procedure

The blood sample was collected from all participants before and 12 weeks after the intervention. 6 mL of blood containing EDTA (Ethylene diamine tetraacetic Acid) and 2 mL without anti-coagulants were obtained. The serum extracted from the blood samples to measuring C-reactive protein (CRP). In contrast, the EDTA-blood samples employed the flow cytometry technique and assessed the ESR.

### Cell Isolation and Flow Cytometry

**Figure 2:**
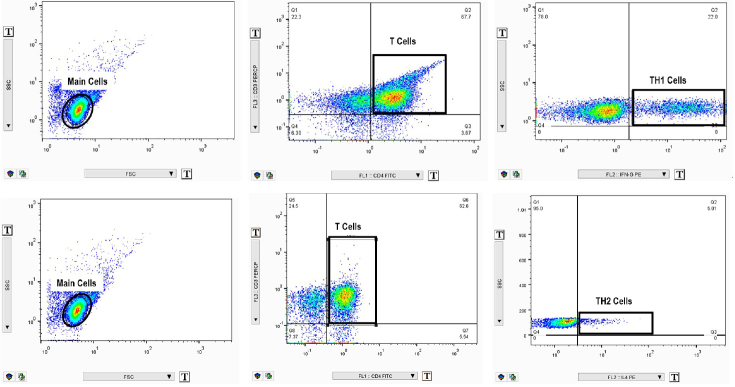
Gate strategy for defining Th1 and Th2 cells. (A) Gating method to identify Th1 cells. Lymphocytes (A1) were gated in SSC and FSC, followed by CD3 against CD4 (A2). CD3^+^CD4^+^ cells were named T cells. Th1 cells were then assessed for IFN-γ production. Finally, CD3^+^CD4^+^IFN-γ^+^ T cells were measured as Th1 (A3) cells. (B) Gating method to identify Th2 cells. Lymphocytes (B1) were gated in SSC and FSC, followed by CD3 against CD4 (B2). CD3^+^CD4^+^ cells were named T cells. Th2 cells were then evaluated for IL-4 presentation. Finally, CD3^+^CD4^+^IL-4^+^ T cells were measured as Th2 (B3) cells.

Ficoll separation was employed to isolate mononuclear cells from the collected blood samples. The viability of these cells was assessed using neobar lam and trypan blue dye. The isolated cells were then cultured and incubated for 5 h at 37°C in an environment containing 5% CO_2_. This incubation aimed to stimulate Th1 and Th2 cells to produce interferon (IFN)-γ and interleukin-4 (IL-4), respectively. The culture medium comprised Roswell Park Memorial Institute Medium (RPMI medium), Pen/Strep, fetal bovine serum (FBS), and the cell activation cocktail with brefeldin (Biolegend, Cat. 423303). For the analysis of Th1 and Th2 cells *via* flow cytometry according to the Biolegend protocol, the cells were stained with fluorochrome-conjugated antibodies, including Fluorescein isothiocyanate (FITC) anti-human CD4 (clone OKT4, Cat. 317408) and PerCP anti-human CD3 (clone SK7, Cat. 344814). After cell fixation and permeabilization, staining with PE anti-human IFN-γ (clone B27, Cat. 506507) and PE anti-human interleukin (IL)-4 (clone MP4-25D2, Cat. 500810) antibodies was carried out for Th1 and Th2 cells, respectively. All samples were analysed with a Partec PAS II Flow cytometer Coulter (PAS, Germany), and the data were processed using Tree Star FlowJo 7.6.1 software and gating strategy presented in Figure 2.

### Data Examination, Statistical Analysis, and Data Interpretation

The outcomes of this study were presented as mean ± standard error of mean (SEM). Statistical analyses were conducted using IBM SPSS version 20, and graphical representations were generated using GraphPad Prism 8.4.3. Parametric and nonparametric data were compared using paired *T*-tests or unpaired *T*-tests and Wilcoxon or Mann-Whitney tests, respectively. A *P*-value of less than 0.05 (*P* < 0.05) was considered statistically significant in all tests.

## Results

### Patient Information

The mean age was 43.73 ± 2.60 in curcumin group and 45.47 ± 2.64 in placebo group. There was no significant difference between the two groups regarding age (*P* = 0.6441), body mass index (*P* = 0.6016), duration of disease (*P* = 0.9105) and DAS28-ESR (*P* = 0.7742) at baseline.

Usage of DMARD was not changed throughout the duration of the study. In the both groups, the average dose of hydroxychloroquine (200 ± 0 mg per day), sulfasalazine (500 ± 0 mg per day), leflunomide (20 ± 0 mg per day) and adalimumab (40 ± 0 mg per 2 weeks) were not different between two groups, as well before and after of the study. As for methotrexate dosage, the curcumin group displayed values of 12.33 ± 1.07 mg/week and 12.17 ± 1.11 mg /week before and after the study (*P* > 0.99). Correspondingly, the placebo group showed methotrexate dosage of 13.33 ± 1.41 mg/week and 13.33 ± 1.41 mg /week before and after the study (*P* > 0.99). The average dose of prednisolone were 4.09 ± 0.38 mg/day and 4.25 ± 0.38 mg/day before and after the study in the curcumin group. Correspondingly, the placebo group showed dose of prednisolone of 4.09 ± 0.38 mg/day and 4.25 ± 0.38 mg/day before and after the study.

### DAS28-ESR Scores, CRP and ESR Levels in RA Participants

Levels of DAS28-ESR, CRP, and ESR in participants with rheumatoid arthritis before and after the intervention are presented on Table 1.

**Table 1:** Levels of parameters in participants with rheumatoid arthritis in the group before and after the intervention and comparison between the two groups.

Clinical and laboratory index	Curcumin group (*n* = 15)			Placebo group (*n* = 15)			Between two groups		

	Before	After	*P* value	Before	After	*P* value	Curcumin	Placebo	*P* value
CRP (mg/dL)	14.79 ± 3.08	7.36 ± 2.23	0.0132	11.25 ± 0.76	13.75 ± 1.26	0.1037	-8.41 ± 2.35	2.38 ± 1.51	<0.0001
ESR (mm/h)	19.87 ± 3.37	16.17 ± 3.40	0.4528	15.33 ± 2.46	19.07 ± 2.79	0.3236	-6.93 ± 1.02	2.46 ± 2.36	<0.0001
DAS28-CRP	3.74 ± 0.22	2.69 ± 0.19	0.0012	3.54 ± 0.21	3.49 ± 0.33	0.9068	-1.05 ± 0.24	0.009 ± 0.26	0.0026
DAS28-ESR	3.70 ± 0.27	2.96 ± 0.22	0.0448	3.80 ± 0.22	3.62 ± 0.35	0.6703	-0.74 ± 0.23	-0.66 ± 0.41	0.5125
Th1 cell (%)	33.24 ± 2.13	28.12 ± 3.05	0.1980	26.55 ± 1.58	40.15 ± 3.63	0.0009	-6.57 ± 4.35	2.89 ± 4.75	0.1532
Th2 cell (%)	6.24 ± 0.69	8.50 ± 0.61	0.0079	6.45 ± 0.62	4.98 ± 0.42	0.0362	4.55 ± 1.14	-2.80 ± 0.87	<0.0001
IFN-γ GMFI	6.18 ± 0.88	5.22 ± 0.092	0.4370	3.39 ± 0.40	7.02 ± 1.90	0.0609	-1.24 ± 1.06	1.71 ± 1.27	0.0840
IL-4 GMFI	4.11 ± 0.29	4.63 ± 0.39	0.2806	3.71 ± 0.44	3.22 ± 0.37	0.4334	-0.11 ± 0.83	-1.34 ± 0.66	0.0795

Data are presented as mean ± SEM. CRP, C-reactive protein; ESR, Erythrocyte sedimentation rate; DAS28-CRP, Disease Activity Score of 28 joints- CRP; DAS28-ESR, Disease Activity Score of 28 joints-erythrocyte sedimentation rate; Th1 cell, T helper1 cell; Th2 cell, T helper2 cell; IFN-γ GMFI, Interferon gamma Geometric Mean Fluorescence Intensity; IL-4 GMFI, Interleukin 4 Geometric Mean Fluorescence Intensity.

The results revealed a reduction in DAS28-ESR scores for both groups. Notably, the decline was statistically significant in the curcumin group (*P* = 0.0448), while it remained nonsignificant in the placebo group (*P* = 0.6703).

The findings of this trial demonstrated a substantial reduction in CRP levels within the curcumin group (*P* = 0.0132), while the placebo group experienced a notable increase in CRP levels (*P* = 0.0411).

As for ESR levels, there was a non-significant decrease in ESR levels within the curcumin group (*P* = 0.4528) and an insignificant increase were observed within the placebo group (*P* = 0.3236).

Complete blood count (CBC), Liver (aspartate Aminotransferase, alanine aminotransferase) and renal (blood urea nitrogen, creatinine) function evaluation were non-significant difference between two groups at baseline and 12 weeks after intervention.

### Flow Cytometry Findings

The results of the study according to the percentage of T cells, IL4 and IFN-γ geometric mean fluorescence intensity (GMFI) are shown in Table 1.

The curcumin group displayed a non-significant decrease in Th1 cell percentage (33.24 ± 2.31 to 28.12 ± 3.05, *P* = 0.1980) after 12 weeks. While a marked increase in Th1 cell was noted in the placebo group (26.55 ± 1.58 to 40.15 ± 3.63, *P* = 0.0009).

The Th2 percentage demonstrated a substantial increase from 6.24 ± 0.69 to 8.50 ± 0.61 in the curcumin group (*P* = 0.0079). A notable reduction in Th2 percentage from 6.45 ± 0.62 to 4.98 ± 0.42 was observed in Placebo group (*P* = 0.0362).

The Th1/Th2 cell percentage in the curcumin group revealed a significant decrease (5.68 ± 0.49 to 4.09 ± 0.39, *P* = 0.0194) and a non-significant increase in the placebo group (3.86 ± 0.55 to 4.98 ± 0.49, *P* = 0.1538). Hence, the curcumin-treated group exhibited a rise in Th2 cell differentiation and a reduction in Th1 cell differentiation.

The GMFI of IFN-γ experienced a non-significant decrease in the curcumin group (5.16 ± 0.64 to 5.22 ± 0.92, *P* = 0.4958) and a non-significant increase in the placebo group (4.12 ± 0.28 to 6.72 ± 1.50 *P* = 0.1523).

IL-4 GMFI did not experience a significant difference by the study’s endpoint (4.11 ± 0.29 *vs*. 4.63 ± 0.39, *P* = 0.2806) in the curcumin group. Additionally, a non-significant decrease in IL-4 GMFI was observed after the intervention (3.71 ± 0.44 *vs*. 3.22 ± 0.37, *P* = 0.4334) in the placebo group.

Morover, no complications and adverse effects were observed in our study.

## Discussion

### Clinical and Para-Clinical Measures

According to the known immune regulatory effect of curcumin on immune system pathways, it seems to be a good candidate as adjunctive treatment in RA disease. To maximize its effectiveness, encapsulating curcumin in nanoformulations holds. In the present study, the effect of Sina-curcumin, a novel curcumin-based nano micelle, was investigated on T-cells function and disease activity in RA patients. Our findings showed, the DAS28-ESR and CRP significantly decrease in curcumin recipients. However, a non-significant decrease in the DAS28-ESR and increase in CRP levels were observed in the placebo group. Regarding ESR level, a non-significant decrease in the curcumin group and a nonsignificant increase in placebo group was seen.

Amalraj *et al*.^[18]^ and Pourhabibi Zarandi *et al*., in separate studies, showed that the administration of curcumin led to reduced levels of ESR and CRP, in contrast to those who received placebo.^[19]^ Corroborating the findings of our study, other studies have shown a noteworthy reduction in CRP, ESR, and DAS.^[11,13,15]^ Javadi *et al*. presented findings indicating positive outcomes, though not statistically significant, regarding changes observed in DAS28 among patients with RA treated with 40 mg nano-micelle curcumin three times per day over 12 weeks. Unlike others, the study showed no significant alteration in ESR values between the two groups of 65 patients assigned either curcumin or placebo treatments.^[17]^ Several studies showed that curcumin has beneficial effects on the clinical symptoms of RA patients, such as joint swelling, pain, and discomfort.^[20,21]^ They suggested using a more appropriate dose of curcumin, its more stable form, and a longer treatment time in future trials.^[4,5,22]^

Collectively, these clinical investigations align with our findings regarding the promising potential of curcumin in decreasing CRP, ESR, and DAS levels, thereby mitigating inflammation among RA patients, all while minimizing adverse effects.

### Flow Cytometry

In the present study, the Th1/Th2 ratio favorably decreased in the curcumin group. This finding was due to a significant increase in Th2 cells and a non-significant decrease in Th1 cells.

Various investigation highlighted a decline in CD4 Tregs count in RA patients. It also noted an altered count of Th2 cells and an uneven distribution of Th subpopulations, heightened Th1 cell presence.^[23,24,25]^ Several animal models and human clinical trials revealed curcumin’s immunomodulatory functions.^[4,26]^ Molazadeh *et al*. revealed inducing Th2 and Treg cell production and boosting IL-10 production from these cells with curcumin.^[26]^ Curcumin-induced immunomodulatory effects were attributed to the deviation of pro-inflammatory Th1 cell responses to anti-inflammatory Th2 cell types, inhibition of pro-inflammatory Th17 cells, and upregulation of Treg cell activity.^[4]^ Regarding the effects of anti-inflammatory phytochemicals in rheumatoid arthritis, Nathan et al suggested curcumin as a more economical and safer therapeutic agent in the treatment of RA.^[27]^

In contrast, Rahimi *et al*. challenged these findings, asserting that curcumin’s anti-inflammatory effects curtailed the differentiation of CD4^+^ Th1, Th2, and Th17 cells while promoting Treg cell induction.^[28]^

## Conclusion

The result of present investigation demonstrated that daily administration of 40 mg Sina curcumin nano formulated exhibit a notable immunoregulatory effects on T-cell function, contributing to a decreases Th1 cells, increases Th2 cells and reduces disease activity in RA patients. Moreover, the findings of the study revealed non significant decrease in IFN-γ GMFI index level and increase the IL-4 GMFI index. According to these cases, curcumin can be used as a promising complementary therapy with substantial anti-inflammatory and immunoregulatory properties for RA patients.

## Limitations of the Study Design

Our study encounters several limitations. The sample size and duration time are small, and a low curcumin dosage utilization. Because our study is the first study to evaluate nano-formulated curcumin in patients with RA, we performed our trial in a pilot format to determine side effects and other probable reactions in our patients. Additionally, no statistically significant differences in the intra-group comparison of dosage before and after treatment cannot be equated to a stable background treatment dosage. Therefore, it is difficult to ensure the balance of random errors between groups. In finally, the study cannot be generalized to all rheumatoid arthritis patients.

## CRediT origin contribution assertion

The clinical study was conducted by Faezeh Khamar. The process of data compilation, statistical analyses, interpretation of findings, and manuscript preparation was undertaken by Faezeh Khamar Under supervision of Mahnaz Sandoughi and Mahdi Atabaki. Patient recruitment and comprehensive medical record maintenance were carried out by Mahnaz Sandoughi. Additionally, Morteza Samadi provided valuable guidance to the research team. The blueprint for the clinical study was developed by Mahdi Atabaki, who oversaw every stage of the research, aided in manuscript refinement, and provided overall research support. All authors participated in the review and analysis of the manuscript.
